# Can Melatonin Be a Potential “Silver Bullet” in Treating COVID-19 Patients?

**DOI:** 10.3390/diseases8040044

**Published:** 2020-11-26

**Authors:** Daniel P. Cardinali, Gregory M. Brown, Seithikurippu R. Pandi-Perumal

**Affiliations:** 1 Faculty of Medical Sciences, Pontificia Universidad Católica Argentina, Buenos Aires 1007, Argentina; daniel_cardinali@uca.edu.ar; 2 Centre for Addiction and Mental Health, Department of Psychiatry, University of Toronto, Toronto, ON M5T 1R8, Canada; gregory.brown@camh.ca; 3 Somnogen Canada Inc., College Street, Toronto, ON M6H 1C5, Canada

**Keywords:** aging, anti-SARS-CoV-2 vaccination, chronotherapy, COVID-19 pandemic, cytoprotection, diabetes, inflammation, metabolic syndrome, melatonin, cognitive impairment, neurodegeneration, oxidative stress, renin–angiotensin system

## Abstract

The therapeutic potential of melatonin as a chronobiotic cytoprotective agent to counteract the consequences of COVID-19 infections has been advocated. Because of its wide-ranging effects as an antioxidant, anti-inflammatory, and immunomodulatory compound, melatonin could be unique in impairing the consequences of SARS-CoV-2 infection. Moreover, indirect evidence points out to a possible antiviral action of melatonin by interfering with SARS-CoV-2/angiotensin-converting enzyme 2 association. Melatonin is also an effective chronobiotic agent to reverse the circadian disruption of social isolation and to control delirium in severely affected patients. As a cytoprotector, melatonin serves to combat several comorbidities such as diabetes, metabolic syndrome, and ischemic and non-ischemic cardiovascular diseases, which aggravate COVID-19 disease. In view of evidence on the occurrence of neurological sequels in COVID-19-infected patients, another putative application of melatonin emerges based on its neuroprotective properties. Since melatonin is an effective means to control cognitive decay in minimal cognitive impairment, its therapeutic significance for the neurological sequels of SARS-CoV-2 infection should be considered. Finally, yet importantly, exogenous melatonin can be an adjuvant capable of augmenting the efficacy of anti-SARS-CoV-2 vaccines. We discuss in this review the experimental evidence suggesting that melatonin is a potential “silver bullet” in the COVID 19 pandemic.

## 1. Introduction

According to Wikipedia, in folklore, a bullet cast from silver is often one of the few weapons that are effective against a werewolf or witch [1]. The term is also a metaphor for a simple, seemingly magical solution to a difficult problem: For example, penicillin was a silver bullet that allowed treatment and successfully cures of many bacterial infections. We discuss in the present minireview the potentiality of melatonin, a molecule of unusual phylogenetic conservation present in all known aerobic organisms, to serve as a preventive and therapeutic agent in COVID-19 pandemic. 

Such a possibility has been the subject of analysis in the literature [2,3]. As an extension to that view, we hereby discuss evidence implying that melatonin (a) prevents SARS-of CoV-2 infection; (b) is suitable as an effective anti-inflammatory/immunoregulatory/antioxidant agent; (c) counteracts chronodisruption; (d) combats several comorbidities such as diabetes, metabolic syndrome, and ischemic and non-ischemic cardiovascular diseases, which aggravate COVID-19 disease; (e) exerts a neuroprotective effect in acutely and chronically affected SARS-CoV-2 patients; and (f) can be an adjuvant to potentiate anti-SARS-CoV-2 vaccines. This multifactorial therapeutic potential is unique to melatonin and is not shared by any other therapeutic drug candidate for the COVID 19 pandemic. The medical literature was identified by searching databases including MEDLINE and EMBASE, bibliographies from published literature, and clinical trial registries/databases. Searches were last updated on November 23, 2020.

## 2. Melatonin in SARS-CoV-2 Infection

The SARS-CoV-2 virus gains entry via the angiotensin-converting enzyme (ACE) 2 in pulmonary epithelial cells and other tissues and organs. The spike glycoprotein on the virion surface docking onto the ACE2 dimer is an essential step in the process of SARS-CoV-2 infection in human cells [4]. Down-regulation of ACE2 expression with systemic renin–angiotensin system imbalance occurs together with the promotion of multi-organ damage.

The ACE2 receptor needs to be within lipid rafts and seems to need to form a dimer for SARS-CoV-2 to gain entry. The trimer of the spike glycoprotein on the virion surface docking onto the ACE2 dimer structure is an essential step in the attack by SARS-CoV-2 on human cells and leads to systemic organ injury [5,6]. After membrane fusion, the viral RNA genome is released into the cytoplasm and is translated into two polyproteins that are cleaved by the SARS-CoV-2 main protease, also called chymotrypsin-like protease, to result in the replication-transcription complex. 

Several experiments suggest that melatonin may be an effective antiviral agent in COVID-19 pandemic (e.g., [7]). By implementing a systems pharmacology-based network medicine platform, quantifying the interplay between the envelope and nucleocapsid proteins of HCoV-host interactome and drug targets in the human protein-protein interaction network, sixteen potential anti-HCoV repurposable drugs were identified, including melatonin, mercaptopurine, and sirolimus [8]. A drug combination of melatonin plus mercaptopurine was identified as effective to hit the HCoV-host subnetwork and was recommended as a potential drug combination to be employed in SARS-CoV-2 infection.

In another study, the structure and physico-chemical properties of melatonin were examined using electronic structure methods and molecular-mechanics tools as a predictor of melatonin’s bioactivity against the coronavirus 2 proteins [9]. Based on the docking scores obtained, the authors proposed that melatonin could be effective to defend against the viral load in vulnerable populations.

The main protease of SARS-CoV-2 is an enzyme conserved among the coronavirus species. By using in silico tools to identify new possible SARS-CoV-2 main protease inhibitors, molecular docking studies described the binding sites and the interaction energies of 74 ligand complexes [10]. Melatonin revealed better interaction energy with the SARS-CoV-2 main protease than the other ligands. 

Another possible way melatonin may regulate viral infection is related to its effective binding and inhibition of calmodulin (CaM) [11,12]. CaM regulates the surface expression and retention of ACE2 in the plasma membrane, and inhibitors of this calcium-binding protein enhance the release of the ACE2 ectodomain by decreasing the association between CaM and ACE2 [13]. Thus, melatonin could be classified as an indirect inhibitor of ACE2-SARS-CoV-2 coupling during viral particle fusion. 

This indirect evidence of possible antiviral action of melatonin may explain the results obtained in a study monitoring 11,672 patients with a statistical model that predicted infection by COVID-19 [14]. Male, African American, older patients, and those with known COVID-19 exposure were at higher risk of being positive for COVID-19, while the risk was reduced in those who were on melatonin, paroxetine, or carvedilol treatment. 

SARS-CoV-2-ACE2 interaction has generated great interest in the development of renin-angiotensin system-based therapeutic strategies for COVID-19. In general, the renin-angiotensin system induces vasoconstriction, hypertension, inflammation, fibrosis, and proliferation via the ACE/angiotensin II/angiotensin II type 1 receptor (AT1R) axis and induces the opposite effects via the ACE2/angiotensin (1–7)/Mas axis function [15,16]. The renin-angiotensin system is activated by chronic inflammation in hypertension, diabetes, obesity, and cancer. SARS-CoV-2 induces ACE2 internalization and shedding, leading to the inactivation of the ACE2/angiotensin (1–7)/Mas axis. It has been hypothesized that two hits to the renin-angiotensin system drive COVID-19 progression in those with pre-existing inflammation. The first hit originates from the chronic inflammation activating the ACE/angiotensin II/AT1R axis, and the second hit originates from the COVID-19 infection inactivating the ACE2/angiotensin (1–7)/Mas axis [17]. These two hits to the renin-angiotensin system could be the primary reason for increased mortality in patients with COVID-19 who have comorbidities with low-degree inflammation such as obesity, diabetes, hypertension, and cancer, or in aged patients. Melatonin is an effective inhibitor of the angiotensin II activation and presumably facilitates angiotensin (1–7) action [18,19,20]. Thus, the two hits to the renin–angiotensin system can both be inhibited by melatonin administration.

## 3. Melatonin as an Anti-Inflammatory/Immunoregulatory and Antioxidant Treatment

Melatonin, a methoxyindole present in all forms of life with aerobic respiration and whose primary function is apparently cytoprotection, has indirect antiviral actions as an anti-inflammatory, antioxidant, and immunoregulatory agent [21,22]. 

### 3.1. Anti-Inflammatory/Immunoregulatory Activity of Melatonin

T lymphocytes are the most evolved cells of the human immune system. T helper lymphocyte (Th) (CD4+) cells typically include Th1, Th2, and Th17 (CD4+CD17+) cells and regulatory T (Treg) (CD4+CD25+) cells. Th1, Th2, and Th17 cells are called effector T cells, relative to Treg cells [23]. Despite the great complexity of the immune system, the foundations of its function are substantially based on three main T lymphocyte subsets, namely Th1, Treg, and T17 lymphocytes. 

Th cells activate T reg lymphocytes [24], inhibit the Th17 cells [25,26], and promote antigen-independent cytotoxicity by inducing the evolution of natural killer (NK) cells into lymphokine-activated killer cells [27,28,29]. The most important actions of Th cells are accomplished by secretion of IL-2, the main growth factor for T lymphocytes [27,30]. 

Relationships occurring among these three major T lymphocyte subsets constitute major biomarkers of the main human systemic diseases, including cancer, autoimmune diseases, and infections. Three relevant ratios include the Th1-to-Treg cell ratio (Th1/Treg R), Th17-to-Treg cell ratio (Th17/T reg R), and Th1-to-Th17 cell ratio (Th1/Th17 R). An abnormally low Th1/Treg ratio is the main characteristic of advanced neoplasms, depending on a decrease in Th1 cell count in association with an increase in T reg cell number [31]. An increase in Th17/Treg R, due to an increase in Th17 cells and a decline in T reg cells inhibited by the action of Th17 cells [26], is the main characteristic of autoimmune diseases. Such an increase in Th17/Treg R occurs also in coronavirus-induced acute respiratory distress syndrome [32,33].

The primary pathophysiology of SARS-CoV-2 infection involves the dramatic upregulation of pro-inflammatory cytokines, induced by the activation of neutrophils, macrophages, and mast cells (“cytokine storm”). It includes increases in interleukins (IL)-1β, IL-6, and IL-17; C-reactive protein; and tumor necrosis factor (TNF) α, and it is usually followed within one week by a gradual increase in levels and activity of the endogenous anti-viral cells, viz CD8+ T cells, NK cells, and γ δ-T cells [34]. However, the activity of this anti-viral response is impaired in SARS-CoV-2 infection, with these suppressed cells showing evidence of exhaustion, which is classically associated with the immune-suppression observed in the tumor microenvironment. 

Melatonin exerts anti-inflammatory effects through various pathways. One of them is sirtuin-1, which inhibits the polarization of macrophages towards the proinflammatory type [35,36]. The anti-inflammatory effect of melatonin also includes the suppression of NF-κB activation [37,38,39]. Moreover, the production of Nrf2 was stimulated by melatonin in hepatoprotection and cardioprotection studies [40]. Inflammation is commonly associated with elevated production of cytokines and chemokines. Melatonin causes a reduction of proinflammatory cytokines (TNF-α, IL-1β, IL-6, L-8, IL-17) and an elevation in the level of anti-inflammatory cytokines such as IL-10 [35,41].

In SARS-CoV-2 infection, hyperinflammatory monocytes/macrophages accumulate in abundance in the lower respiratory tract, where they play a key role in determining the severity of the disease. Monocytes/macrophages infected with SARS-CoV-2 virus reprogram their metabolism from mitochondrial oxidative phosphorylation to the cytosolic glycolysis for ATP production (Warburg effect) via generation of reactive oxygen species that stabilize hypoxia inducible factor-1α (HIF-1α) [42]. Monocytes/macrophages functioning with this metabolic phenotype produce more cytokines, leading to T cell destruction and killing of the alveolar lining cells, severely aggravating the COVID-19 infection. Melatonin converts highly pro-inflammatory glycolytic M1 macrophages to anti-inflammatory M2 macrophages, which utilize mitochondrial oxidative phosphorylation [43]. This effect of melatonin may be exerted via the well-documented down-regulation of HIF-1α [36].

### 3.2. Antioxidant Properties of Melatonin

In both the cytoplasm and the cell nucleus, melatonin has important antioxidant and scavenging effects on free radicals, which are largely independent of receptors [41]. These effects are exerted in three ways: (a) melatonin is a free radical scavenger; (b) melatonin is metabolized to compounds with high antioxidant activity; and (c) melatonin is an indirect antioxidant, which stimulates the synthesis of antioxidant enzymes and inhibits that of prooxidant enzymes. Melatonin has a proven superiority to vitamin C and E in protection against oxidative damage and the elimination of free radicals [44]. In addition, melatonin potentiates the effects of other antioxidants, such as vitamin C and Trolox. Several antiapoptotic and cytoprotective effects of melatonin are exerted under conditions of ischemia (unrelated to free radicals) and can be attributed to its stabilizing action on the mitochondrial membrane [45].

In diseases showing a high level of inflammation, the application of melatonin showed promising results with strong attenuation of circulating cytokine levels. This was documented in patients with diabetes mellitus and periodontitis [46] and severe multiple sclerosis [47]. Moreover, in the acute phase of inflammation, during surgical stress [48], cerebral reperfusion [49], or reperfusion of the coronary artery [50], treatment with melatonin reduced the level of proinflammatory cytokines. 

Generally, these anti-inflammatory/immunoregulatory and antioxidant effects of melatonin need doses as calculated by allometry that are well above the 3–10 mg/day range in which melatonin exerts chronobiotic effects. Allometry applies to properties whose proportions change as a function of size, as opposed to isometry whose relationship to size remains constant. Body surface area, rather than body weight, correlates well across several mammalian species with several parameters of biology, including oxygen utilization, caloric expenditure, basal metabolism, blood volume, circulating plasma proteins, and renal function, and has been advocated as a factor to be used when converting a dose for translation from animals to humans [51]. Allometry is commonly used for determining doses for Phase I human clinical drug trials. In clinical medicine, it has been feasible to convert adult data by allometry to predict drug pharmacokinetic parameters in children, which can significantly decrease the occurrences of toxicity and mortality for new drugs used in children. Noteworthily, theoretical human equivalent doses calculated from animal studies examining the anti-inflammatory/immunoregulatory/antioxidant activity of melatonin ranged from 2- to 3-orders of magnitude greater than those usually employed in humans, i.e., in the 100–300 mg/day range [52].

According to the COVID-19 clinical reports, patients with a severe infection have an increased risk of sepsis and cardiac arrest [53,54]. The available information indicates that the application of melatonin can improve septic shock through inhibition of the NLRP3 pathway [55]. In rats, melatonin has a preventive effect against sepsis-induced kidney damage, septic cardiomyopathy, and liver damage [56,57,58]. In human neonatal sepsis, the improvement of clinical outcome after melatonin treatment was documented [59,60,61,62]. Melatonin has also been reported as beneficial in patients with myocardial infarction, cardiomyopathy, hypertensive heart disease, and pulmonary hypertension. In critically affected patients, deep sedation is associated with increased long-term mortality, and the application of melatonin reduces the use of sedation and the frequency of pain, agitation, and anxiety [63] and also improves the quality of sleep in intensive care unit patients. Therefore, the rationale for the use of high doses of melatonin in COVID-19 focuses not only on the attenuation of infection-induced respiratory disorders but also on general improvement and prevention of possible complications, including neurologic complications [64].

A recent study determined the efficacy and tolerability of high-dose melatonin (36 mg/day to 72 mg/day p.o. in four divided doses) as adjuvant therapy, in addition to standard and/or empirical therapy [65]. All the patients were admitted with flu-like symptoms and chest imaging findings of ground-glass opacities highly suggestive of COVID-19 pneumonia. The 10 patients given melatonin had high-risk features determined for age (>60 years) or/and established comorbidities. No significant side effects were noted except for drowsiness. Benefits of time for clinical improvement (reduction of symptoms, stabilization and/or regression of lung infiltrates, decrease in proinflammatory markers) were observed, as well as the need for mechanical ventilation, duration of hospital stay, and outcome (death, or recovery and discharge) [65].

Another recent report was a retrospective analysis based on the clinical experience at the Columbia University Irving Medical Center related to drugs used to treat respiratory distress in COVID-19-infected patients who required endotracheal intubation [66]. After a comprehensive evaluation of 791 patients diagnosed with COVID-19 who required intubation, the application of melatonin was the only drug that was statistically associated with higher positive clinical outcomes, including survival in intubated patients as well as in those requiring mechanical ventilation. As of the present date November 23, 2020, this paper is published as a preprint [66].

## 4. Melatonin as a Chronobiotic Agent

The term chronobiotic was introduced in the early 1970s and has been used to broadly define a drug that affects the physiological regulation of the body clock and, specifically, one that is capable of therapeutically recovering desynchronized circadian rhythms in the short or long term, or prophylactically avoiding its interruption after an environmental attack [67]. The magnitude and direction of phase changes depend on the circadian phase in which the compound is administered, which in turn produces pronounced phase changes in behavioral rhythms. For example, melatonin given in the morning delays the phase of circadian rhythms, while when given in the evening it advances the phase of circadian rhythms. For most of the day, melatonin administration is unable to modify the phase of the endogenous clock (phase-response curve) [68]. 

The association of aging with a higher vulnerability to COVID-19 infection is a subject of major importance. Several factors, including higher stress due to social isolation, diminished melatonin levels with age, and inadequate exposure of individuals to light in the evening, which reduces melatonin levels and disrupts circadian rhythmicity, are important for maintaining the circadian health in aged individuals. Among several other comorbidities, the aged population is more prone to suffer from coronavirus infection, and the association of aging with a higher vulnerability to COVID-19 infection is currently a subject of major importance [33]. Increased stress and depression in socially isolated seniors lead to increased proinflammatory and decreased anti-viral immune responses. Among the consequences of staying indoors during the forced lockdown period, disruption of circadian rhythmicity, particularly of the sleep/wake cycle, is highly frequent, and age-associated circadian misalignment develops. Dysregulation of circadian timing systems is thought to be involved in several medical and mental conditions in aged individuals, especially cardiovascular and neurodegenerative diseases [69,70]. 

Forced lockdown such as during the current pandemic disrupts timing and duration of exposure to ambient light, the most important environmental Zeitgeber. The use of mobile phones, tablets, and computers to watch the news, binge-watching of web series, and connecting on social media leads to excessive screen time in evening hours. The blue light emitted from screens suppresses the natural production of melatonin at night. Activity levels during the day also influence the sleep pattern; low levels of activity (whether due to confinement or depression) negatively affect sleep, as does strenuous activity (e.g., due to stress or work overload) [71,72].

Aging often is associated with a significant reduction in sleep efficiency and continuity, and this coincides with a significant reduction in amplitude of the melatonin rhythm and consequently of many other circadian rhythms as well [73]. An increase in early morning awakenings and difficulty in falling asleep have been frequently reported in the elderly. Impaired melatonin secretion is associated with sleep disorders that are encountered in elderly insomniacs. Indeed, aging may be a process resulting from, or aggravated by, the relative circadian desynchrony produced by melatonin deficiency. Melatonin can be effective for improving the quality of life in the elderly via its recognized chronobiotic capacity [74].

The common causes of sleep disturbance during aging include, but are not limited to, lifestyle (e.g., retirement life), pre-existing medical and mental illnesses, polypharmacy, poor sleep habits, pre-existing sleep disorders, and psychological distress [75]. Sleep dysfunctions and sleep disorders are highly prevalent in the aging population [71]. As mentioned above, sleep disruption has become more prevalent during the COVID-19 pandemic. 

The objective of chronotherapy is to optimize medical treatments, taking into account the body’s circadian rhythms [76,77]. Chronotherapy works via two means: (a) it alters the sleep/wake rhythms of patients to improve the sequels of several pathologies; and (b) improved timing of therapies can be achieved by evaluation of the circadian rhythms of patients. Both approaches are relevant for implementation of chronotherapeutic strategies in aged individuals during the COVID-19 pandemic.

Even minor dysfunctions of the biological clock can greatly affect sleep/wake physiology, causing excessive diurnal somnolence, increase in sleep onset latency, phase delays or advances in sleep onset, frequent night awakenings, reduced sleep efficiency, delayed and shortened rapid eye movement sleep, or increased periodic leg movements [78]. Chronotherapy is designed to restore the proper circadian pattern of the sleep-wake cycle in the elderly through adequate sleep hygiene, timed light exposure, and the use of a chronobiotic medication like melatonin, which affects the output phase of circadian rhythms, thus controlling the clock [79].

Concerning the second basis of chronotherapy, it should be stressed that the immune system displays very strong circadian rhythmicity [80]. At the beginning of daily activity, there is increased expression of pro-inflammatory mediators such as interleukin (IL)-1β, IL-6, and IL-12, as well as macrophage and leukocyte activity, which leads to potential damage to tissues. By contrast, anti-inflammatory mediators and other growth or angiogenesis factors peak during the resting phase (see, for example, [81]). Both CD4+ and CD8+ T cell activities against viral antigens reach their highest levels during the resting phase, while the cytotoxic activity of natural killer cells is most severe at the beginning of the active part of the day. 

Indeed, the time of day in which a viral infection occurs affects survival. For instance, infections at the beginning of the activity phase are more fatal than infections that occur at the beginning of the resting phase [82]. These temporal patterns may be disrupted in aged individuals, and thus circadian disorganization should be taken into account when using immune modulators and anti-inflammatory agents in the older population [81]. It is plausible that proper circadian timing of anti-inflammatory drugs (chronotherapy) can target the detrimental inflammatory cascade in COVID-19 patients without interfering with the fight of the immune system against the virus. This can be extremely important for low dose dexamethasone treatment, given the recent demonstration that it may reduce mortality in severely-infected COVID-19 patients to one-third [83].

Delirium is found in up to 50% of hospitalized elderly patients and 80% of critically ill patients who receive mechanical ventilation [84]. Treatment of this chronodisruption with melatonin is associated with a shortened intensive care unit stay, reduced prevalence of delirium, and improved sleep quality [85]. In COVID-19 disease, about 15% of hospitalized patients show impaired consciousness ranging from somnolence to confusion, delirium, stupor, and coma [86]. Melatonin should be considered as an agent effective in improving sleep and with the potential to minimize the administration of benzodiazepines or antipsychotics that could worsen delirium in the elderly or those with central respiratory depression [87].

## 5. Melatonin and Cytoprotection

Diabetes mellitus, metabolic syndrome, and ischemic and non-ischemic cardiovascular diseases are comorbidities that aggravate COVID-19 disease. The prevalence of metabolic syndrome varies from 15 to 30% depending on the region of the world considered, and an increase of 1.5 to 2.5 times in cardiovascular mortality occurs when the metabolic syndrome is present [88,89]. As reported by the Centers for Disease Control and Prevention, USA, it is estimated that individuals with metabolic syndrome following diabetes mellitus type 2 might have up to ten times greater risk of death due to COVID-19 [90]. The number of identified cardiovascular comorbidities in confirmed COVID-19 cases varied from 4.2% to 40%, and the incidence of acute cardiac injury in the course of the disease ranged from 12% to 23% according to illness severity of COVID-19-patients investigated [91]. Thus, an adequate control of these diseases is a major goal to achieve in the ongoing pandemic.

In humans, circulating melatonin levels are consistently reduced in diabetes, metabolic syndrome, and ischemic and non-ischemic cardiovascular diseases, and the therapeutic value of melatonin has been suggested by a limited number of clinical trials generally employing melatonin in the 2–5 mg/day range [92,93]. In animal model studies of the metabolic syndrome, and ischemic and non-ischemic cardiovascular diseases, melatonin was very effective in curtailing symptomatology [52]. Almost every cell in the human body contains melatonin in quantities much higher than those circulating in blood-derived from the pineal gland [94]. The mitochondrial capacity to synthesize melatonin has now been confirmed, but for reasons that remain unexplained, intracellular melatonin does not enter the extracellular space. To modify intracellular melatonin levels, doses much higher than those employed as a chronobiotic are needed [95]. Moreover, allometric calculations derived from animal studies indicate projected cytoprotective melatonin doses for humans in the 40–100 mg/day range, doses that are rarely employed in clinical practice.

## 6. Melatonin and Neuroprotection

In patients with severe COVID-19 disease, neurological complications comprising anosmia, stroke, paralysis, cranial nerve deficits, encephalopathy, delirium, meningitis, and seizures have been documented (see, for example, [64,96]). It remains to be established whether neurological abnormalities are caused by SARS-CoV-2 itself, by the exaggerated cytokine response it triggers, and/or by the increased formation of blood clots in brain blood vessels. In patients with neurological symptoms, augmented cerebrospinal fluid autoantibodies [97], white matter change in the brain [98,99], and psychological and psychiatric consequences occur [100]. 

**Figure 1:**
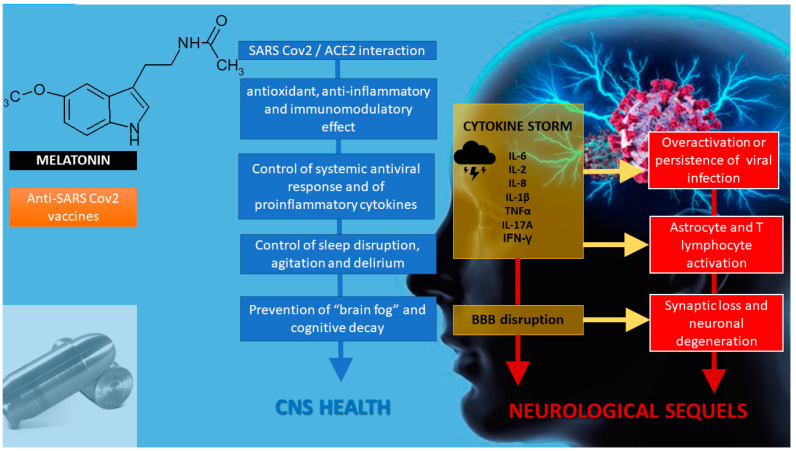
Melatonin as a potential “silver bullet” in the COVID 19 pandemic, as exemplified in the brain. Melatonin has possible antiviral activity by interfering with SARS-CoV-2/angiotensin-converting enzyme 2 association. As an antioxidant, and anti-inflammatory and immunomodulatory compound, melatonin impairs the consequences of SARS-CoV-2 infection. Melatonin is an effective chronobiotic agent that reverse circadian disruption and delirium in intensive care unit patients. Melatonin may prevent neurological sequelae in COVID-19-infected patients like “brain fog” and cognitive decay. Melatonin can be an adjuvant for augmenting the efficacy of anti-SARS-CoV-2 vaccines. BBB: blood brain barrier.

In a recent study, to the present date (23 November 2020) published as a preprint, cognitive test data were obtained from 84,285 Great British Intelligence Test participants who completed a questionnaire regarding suspected and biologically confirmed COVID-19 infection [101]. People who had recovered, including those no longer reporting symptoms, exhibited significant cognitive deficits when controlling for age, gender, education level, income, racial-ethnic group, and pre-existing medical disorders. The scale of the observed deficits was equivalent to an average 10-year decline in global performance between the ages of 20 to 70 within this dataset. As a comparison, the authors remarked that this deficit was larger than the mean deficit of 512 people who indicated they had previously suffered a stroke and 1016 who reported learning disabilities [101]. “Brain fog”, i.e., confusion, forgetfulness, inability to focus, fatigue, and low mental energy [102,103] may be thus an emerging major sequela of COVID-19 infection (Figure 1).

In this context, the neuroprotective properties of melatonin deserve consideration [104]. An analysis of published data using melatonin in the early stages of cognitive decline consistently showed that administration of melatonin, every night before retiring, improves the quality of sleep and cognitive performance disease [105]. Patients treated with melatonin showed significantly better performance in various neuropsychological tests. They also had lower scores in the Beck Depression Inventory concomitantly with improvement in the quality of sleep and wakefulness [106]. 

Retinal cells are particularly vulnerable to neurodegeneration. For example, in patients with Parkinson’s disease, a thinner lamina cribrosa (the site where optic nerve axons of the retina run) has been documented [107]. A recent study [108] explored the effect of melatonin treatment on lamina cribrosa thickness in patients with minimal cognitive impairment. After 6 months, treatment with 0.15 mg/kg melatonin significantly augmented lamina cribrosa thickness and hippocampal volume, decreased CSF tau levels, and improved the mini mental score as compared to the untreated group. Collectively, the data support the effectiveness of melatonin to curtail brain degeneration and underline its therapeutic significance in the neurological sequelae of COVID-19. 

## 7. Melatonin as an Adjuvant in Anti-SARS-CoV-2 Vaccination

Many pharmaceutical companies are now working hard to produce safe and effective vaccines against SARS-CoV-2. However, even if such a vaccine is established, vaccine efficacy may be inferior for the elderly and other high-risk population groups compared to people who are healthy and young. Melatonin may help to close the gap in this respect [109]. 

The first evidence that melatonin could increase the IgG antibody response and counteract the immunosuppressive effect of corticosteroids and/or acute stress was provided by [110]. Melatonin is effective in counteracting the immunosuppression observed in aging [111,112]. This effect of melatonin was linked to an increase in CD4+ T lymphocytes [113]. 

Concerning vaccines, several studies have shown that exogenous melatonin acts as an adjuvant improving the CD8+ T cell response in cancer vaccines [114,115] and also humoral responses against a variety of pathogens [116]. Melatonin enhances the immune response to vaccines by increasing peripheral blood CD4+ T cells and IgG-expressing B cells. These findings are particularly interesting because a recent study in convalescent COVID-19 patients found a vigorous response of CD4+ T cells to the spike protein, the main target of most vaccines, and also that such response was correlated with the level of anti-SARS-CoV-2 IgG and IgA [117]. However, in many patients, the immune response may not be have been sufficient, as relapses have already been reported, so it appears that long-term natural immunity may not prevent current and future flare-ups [118].

Therefore, the development of an effective vaccination is imperative to control the disease. COVID-19 patients, particularly the elderly group, show a decrease in the number of CD8+ T cells due to inhibition of IL-2 and IL-2 receptors. Melatonin is known to stimulate IL-2 production, and by doing this, CD4+ T cells increase [119]. Therefore, its use in vaccination against SARS-CoV-2 can enhance the type of immunity that is most effective against the virus.

Administration of exogenous melatonin could increase the potency of the immune response and the duration of the immunity induced by the vaccine. Moreover, due to its antioxidant properties and its pleiotropic effect on the immune system, melatonin could also prevent the adverse effects of the vaccine [109].

## 8. Concluding Remarks

Unquestionably, the current COVID-19 pandemic is the most devastating event in recent history. The virus causes relatively minor damage to young populations but imposes life-threatening danger to the elderly and people with chronic inflammatory diseases. Young people do not suffer from COVID-19 as much as the elderly, among other causes because they have much higher circulating melatonin levels. 

**Figure 2:**
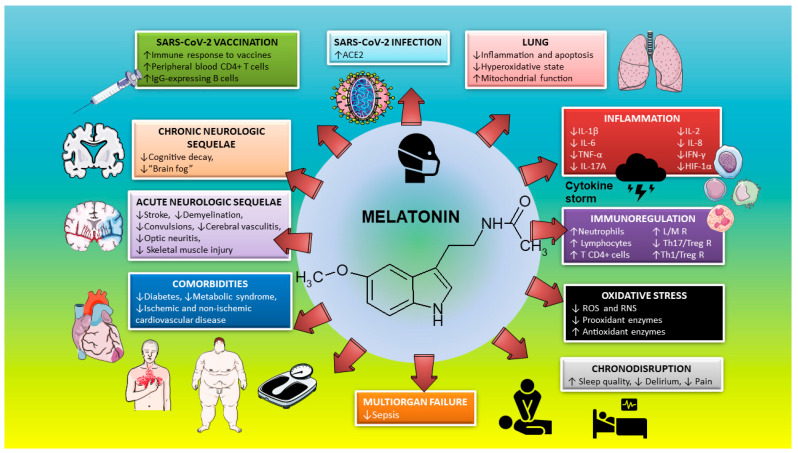
Melatonin as a multifactorial therapeutic agent in SARS-CoV-2 infection. For explanation, see text. ROS: radical oxygen species; RNS: radical nitrogen species. L/M R: lymphocyte/monocyte ratio.

Viruses induce an outstanding increase in inflammatory cytokines and reactive oxygen species, and melatonin, the best natural antioxidant-anti-inflammatory-cytoprotector, has very low levels in aged patients [34]. General immunity is impaired in severely compromised COVID-19 patients, and melatonin stimulates immunity. Therefore, the use of the very safe drug melatonin in adequate doses can prevent the development of severe disease symptoms in coronavirus patients, reduce the severity of their symptoms, and/or reduce the immuno-pathology of coronavirus infection on patients’ health after the active phase of the infection is over. In addition, melatonin may help to reduce reinfections and serve as a powerful immunopotentiating adjuvant for future vaccines (Figure 2).
